# Randomized clinical trial of the effects of screening and brief intervention for illicit drug use: the life shift/shift gears study

**DOI:** 10.1186/1940-0640-9-8

**Published:** 2014-05-22

**Authors:** Susan I Woodruff, John D Clapp, Kimberly Eisenberg, Cameron McCabe, Melinda Hohman, Audrey M Shillington, C Beth Sise, Edward M Castillo, Theodore C Chan, Michael J Sise, Joey Gareri

**Affiliations:** 1 San Diego State University, School of Social Work, Center for Alcohol and Drug Studies, 5500 Campanile Drive, San Diego, CA HH-203E, USA; 2 The Ohio State University, College of Social Work, Columbus, OH 43210-1162, USA; 3 San Diego State University Research Foundation, 6386 Alvarado Ct Ste 224, San Diego, CA 92120, USA; 4 Colorado State University, School of Social Work, 127 Education, 1586 Campus Delivery, Ft. Collins, CO 80523-1586, USA; 5 Scripps Mercy Hospital, Trauma Research & Injury Prevention, Trauma Service [MER-62], 4077 Fifth Ave, San Diego, CA 92103, USA; 6 Department of Emergency Medicine, University of California San Diego, 200 West Arbor Drive, San Diego, CA 92103, USA; 7 University of Toronto, Motherisk Laboratory, The Hospital for Sick Children, 555 University Ave, Toronto M5G 1X8, Ontario,Canada

**Keywords:** Screening and brief intervention, Drug use, Emergency department patients

## Abstract

**Background:**

Although screening, brief intervention, and referral to treatment (SBIRT) has shown promise for alcohol use, relatively little is known about its effectiveness for adult illicit drug use. This randomized controlled trial assessed the effectiveness of the SBIRT approach for outcomes related to drug use among patients visiting trauma and emergency departments (EDs) at two large, urban hospitals.

**Methods:**

A total of 700 ED patients who admitted using illegal drugs in the past 30 days were recruited, consented, provided baseline measures of substance use and related problems measured with the Addiction Severity Index-Lite (ASI-Lite), and then randomized to the *Life Shift* SBIRT intervention or to an attention-placebo control group focusing on driving and traffic safety (*Shift Gears).* Both groups received a level of motivational intervention matched to their condition and risk level by trained paraprofessional health educators. Separate measurement technicians conducted face-to-face follow-ups at 6 months post-intervention and collected hair samples to confirm reports of abstinence from drug use. The primary outcome measure of the study was past 30-day drug abstinence at 6 months post-intervention, as self-reported on the ASI-Lite.

**Results:**

Of 700 participants, 292 (42%) completed follow-up. There were no significant differences in self-reported abstinence (12.5% vs. 12.0% , p = 0.88) for *Life Shift* and *Shift Gears* groups, respectively. When results of hair analyses were applied, the abstinence rate was 7 percent for *Life Shift* and 2 percent for *Shift Gears* (p = .074). In an analysis in which results were imputed (n = 694), there was no significant difference in the ASI-Lite drug use composite scores (*Life Shift* +0.005 vs. *Shift Gears* +0.017, p = 0.12).

**Conclusions:**

In this randomized controlled trial, there was no evidence of effectiveness of SBIRT on the primary drug use outcome.

**Trial registration:**

ClinicalTrials.gov NCT01683227.

## Background

The San Diego-Mexico border is one of the most active drug smuggling corridors in the world. San Diego has been designated as a High-Intensity Drug Trafficking Area due to the large quantities of cocaine, heroin, and methamphetamine that are transported to the County from Mexico. Nearly two-thirds of women and more than half of men arrested and booked into jail for crimes in San Diego County in 2010 tested positive for illicit drugs, such as marijuana, methamphetamine, cocaine, and heroin [1]. It is estimated that the total economic cost of alcohol and drug abuse in the region is more than $240 billion annually, with about $97 billion due to drug abuse [2]. Patients who visit hospital emergency departments (EDs) may be at particularly high risk for a variety of behavioral risk factors such as illicit substance use [3].

Screening, Brief Intervention, and Referral to Treatment (SBIRT) is a comprehensive, integrated public health approach for providing a spectrum of early detection and intervention services for substance use in general medical care settings, including the ED [4,5]. These settings offer a potential “teachable moment” because patients may have perceptions of vulnerability about their health, regardless of the reason for the visit, and therefore may be particularly receptive to screening and counseling [6]. Unlike primary prevention that targets non-risk or low-risk users, or treatment services for people already dependent, SBIRT provides early intervention services targeted at individuals who misuse alcohol or illicit drugs, but who may not be dependent. Although individual program frameworks vary, all SBIRT programs share two key components: screening and intervention. Individuals who screen positive for alcohol or drug problems are provided with an appropriate educational or therapeutic service. Most of those screening positive are categorized as relatively low risk and receive a brief intervention, consisting of a time-limited motivational interview done on site that focuses on increasing patient awareness of the risks of substance abuse, feedback on normative use and safe limits, and eliciting motivation to change [4]. Individuals at moderate- to severe risk are provided brief intervention plus brief treatment (e.g., six face-to-face counseling sessions) or referral to specialty treatment for more intensive support [4].

Although the SBIRT approach has shown promise for alcohol use [7-9], relatively little is known about its effectiveness for illicit drug use specifically [10]. An international study reported that brief intervention in primary health-care settings was associated with reductions in self-reported illicit substance use in several countries, with the exception of the United States [11]. Madras and colleagues [12] found a 68 percent reduction in self-reported illicit drug use among those exposed to screening and brief intervention services, although their study did not include a control group. A randomized study of opioid and cocaine users screened by peer interventionists during an urgent care visit reported a salutary effect of screening and brief intervention on drug use [13]. With the exception of the Bernstein et al. study [13], methodological issues, such as lack of biological confirmation of drug use, short follow-up periods, lack of control groups, and the inability to rule out reactivity to measurement, limit conclusions about intervention effectiveness.

SBIRT is quickly becoming a recommended best practice in a variety of settings, especially in EDs and trauma centers, and billing for SBIRT services is becoming easier as more states activate billing codes. However, rigorous research is needed before SBIRT for drug use is ready for broad universal dissemination [14]. The present study is one of the first rigorous studies to evaluate the effectiveness of SBIRT for illegal drug use. This randomized controlled trial assessed the effectiveness of the SBIRT approach for outcomes related to drug use among patients visiting EDs at two large, urban, acute-care hospitals in Southern California.

## Methods

### Design overview

This study evaluated the effectiveness of SBIRT for drug use and related factors for 700 multi-ethnic trauma and ED patients using a two-group, randomized, repeated-measures design. Self-reported drug use, biologically validated drug use abstinence, health-care utilization, medical and psychiatric problems, and alcohol use in the drug-based intervention group were compared to that of an attention-placebo control group that received equal intervention in the areas of driving and traffic safety. Three bilingual/bicultural health educators (HEs) recruited participants who reported past 30-day illicit drug use that was more severe than their alcohol use from trauma units and the ED waiting areas of two large hospitals (details regarding HE selection and training are described in Eisenberg and Woodruff, 2013 [15]). Following consent procedures and standardized baseline assessments, HEs randomly assigned participants to one of the two conditions. The intervention group received “*Life Shift*”, an SBIRT drug use intervention matched to the participant’s drug risk level. The control group received the same exposure to intervention in an unrelated area—driving and traffic safety (“*Shift Gears*”) that was matched to their driving/traffic risk level. Measurement technicians (MTs)—separate from the interventionist staff and blind to the participants’ assigned conditions—conducted face-to-face, 6-month, follow-up visits and collected the same outcome measures, as well as hair samples for validating self-reported drug abstinence. Approvals from the San Diego State University IRB and hospital IRBs were obtained prior to contact with participants. Trial registration with ClinicalTrials.gov was not completed until all participants were enrolled and follow-up was completed.

### Participant enrollment

Participant enrollment and intervention was conducted from April 2010 to June 2011 in the EDs and trauma units of two large urban hospitals in Southern California. HEs attempted to approach all capable adult patients, regardless of the reason for the patient’s visit. Patients under the age of 18, those with severely altered mental status, those physically incapable of participating due to severe illness or injury, those without any phone number where they could be reached, and those unable to speak English or Spanish were excluded from participation. Further eligibility was then based on responses to two pre-screen items that assessed any current (i.e., past 30 day) alcohol use and use of non-prescribed drugs. The specific screening questions were: “How many times in the past 30 days have you used any alcohol?” and “How many times in the past 30 days have you used any non-prescribed drugs?” Hair samples to confirm self-reported drug use were not collected at baseline, as participants were not expected to falsely report using drugs [16]. Distracter items assessing nutrition, exercise, and driving/traffic safety were asked so that potential participants did not guess the primary purpose of the study. Those answering “0 times” to both the current use of alcohol and drug questions were verbally reinforced and thanked for their interest. Those reporting alcohol use only were given an alcohol use brochure that described lower-risk drinking limits; were encouraged to seek further assistance via the internet and community resources for alcohol problems; and were thanked for their interest. Those reporting illicit drug use were further considered for inclusion.

A sizable number of drug users were also users of alcohol, and depending on the severity of alcohol use, it may pose more harm to the individual than drug use [17]. Researchers were interested in enrolling patients whose illicit drug use severity was equal to or exceeded their alcohol use severity, yet ethical considerations demanded that researchers appropriately address the more severe problem. Both drug and alcohol risk levels were determined for this set of patients using two validated and widely used brief screeners available in both English and Spanish interview formats: a) the AUDIT — Alcohol Use Disorders Identification Test [18], and b) the DAST-10 — Drug Abuse Screening Test [19,20]. HEs computed AUDIT and DAST scores, and applied standard cut-points to determine risk categories (i.e., low risk, at risk, high risk, and severe risk). The AUDIT alcohol risk categories were based on Babor et al. [18] and included no/low risk (score of 0–7), at risk (score of 8–15), high risk (score of 16–19), and severe risk (score of 20–40). The DAST-10 categories were based on those of the test’s developer [20] and included no/low risk (score of 0), at risk (score of 1–2), high risk (score of 3–8), and severe risk (score of 9–10). Patients whose alcohol use risk category exceeded their drug use risk category were given a brochure that described safe drinking limits and were provided internet and community resources for alcohol problems. Those patients whose drug use risk category was equal to or higher than their alcohol risk category were considered eligible, and were then asked if they would like more information about the study. Potential participants were offered $5 on the day of enrollment and baseline assessment, and $20 after completion of a 6-month follow-up interview.

**Figure 1:**
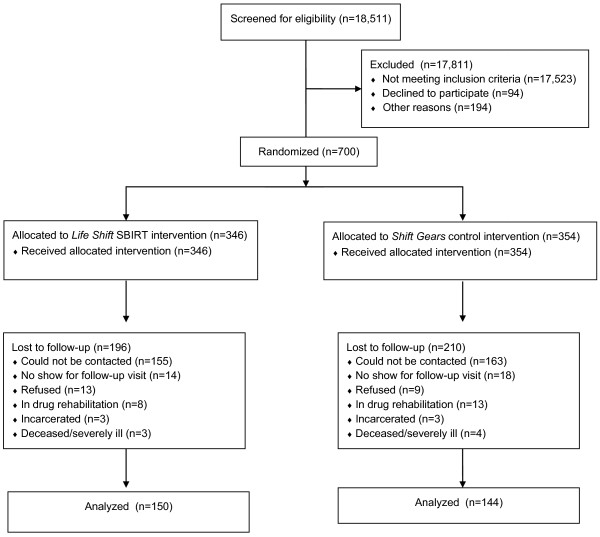
CONSORT diagram showing the flow of patients through the trial.

Figure 1 presents a CONSORT diagram of the flow of patients through the trial [15,21]. Eligibility and interest in participating was assessed for over 18,000 patients. About 95 percent of those approached were not eligible primarily because their drug use risk did not exceed their alcohol use risk. Of the 988 eligible, 9.5 percent declined to participate, and 19.4 percent did not finish the consent process or did not provide the necessary contact information because of interruptions for care from hospital staff. The result was 700 patients enrolled in the study.

### Baseline procedures

An informed consent process was administered to interested, eligible participants. Using an interview format, HEs then collected demographic information; five components of the Addiction Severity Index Lite [22]; a driving and traffic safety measure that was immediately scored into risk categories; and a drug avoidance self-efficacy measure. Participants were then randomly assigned based on last digit of their telephone number to *Life Shift* (even number) or to *Shift Gears* (odd number), and a level of motivational intervention matched to each participant’s condition and risk level was delivered. Because the HE delivered the type and level of intervention, he/she was aware of the participant’s assigned condition.

### Interventions

The *Life Shift* drug intervention and the *Shift Gears* driving/traffic safety intervention were manual-driven, designed to be of the same duration, and were available in English and Spanish. Both had core SBIRT elements, which all participants received, as well as adaptive elements that allowed the HE to tailor the program based on the participant’s needs and risk level [23]. Both interventions began with the HE assessing the participant’s risk level. Interventions were delivered in a non-judgmental manner using a brochure to help communicate the short- and long-term health, social, and legal effects. (For the *Life Shift* intervention, drug-specific brochures were used when marijuana, cocaine, and methamphetamine were the drugs of choice; otherwise, a general drug use brochure was used.) The participant’s motivation or readiness to change, a construct based on the Transtheoretical Model [24], was then assessed using a 4-point scale. The *Life Shift* participants were asked, “How ready are you at the present time to change your use of < specific drug > ?” while the *Shift Gears* participants were asked, “How ready are you at the present time to change your risky driving behaviors?” Participants answered “not at all ready to change”, “slightly ready”, “moderately ready”, or “very ready to change”. The readiness information was used to guide additional discussion about the participant’s drug use (or risky driving/traffic behaviors). Together, the HE and participant made a plan specific to the participant, based on the discussion and recorded ideas on the worksheet on the back of the brochure. A core element of both interventions, and SBIRT in general, was motivational interviewing (MI), a non-confrontational style of behavior change counseling [25,26]. More information about both interventions is described below.

### Life Shift drug use intervention

Based on DAST-10 categories resulting from standard scoring procedures [20] and the MI process, participants in the *Life Shift* group received one of three tracks tailored to their specific risk category:

1. Brief intervention for at-risk individuals scoring 1 to 2 — Brief intervention with at-risk participants was delivered on-site in the ED/trauma unit. This interaction between the participant and HE included feedback, exploring the participant’s ambivalence about drug use and change, and a negotiation/commitment to abstain or reduce use.

2. Brief treatment for high-risk individuals scoring 3 to 8 — In addition to the on-site brief intervention, high-risk participants were offered 6 individual sessions with a *Life Shift* substance use counselor (a licensed therapist) over the phone. The sessions incorporated principles of MI and cognitive-behavioral therapy. The HE offered to make the first phone appointment for the participant.

3. Referral to treatment for severe-risk individuals scoring 9 to 10 — In addition to the on-site brief intervention and the offer of brief treatment phone counseling, severe-risk participants were given a list of local agencies that could provide further assessment and support.

### Shift Gears driving and traffic safety intervention

Participants assigned to the *Shift Gears* program received an appropriate intervention for their specific risk level, based on a modified version of the Driver Behavior Questionnaire (DBQ) [27-29] adapted for our use, and on their readiness to change. The DBQ is an assessment tool designed to identify and classify driving behaviors into specific categories. Levels of *Shift Gears* were two-tiered instead of three-tiered, based on our formative work that showed less variability in driving/traffic risk than drug use risk. However, the intervention was of the same duration (about 15–20 minutes for a brief intervention) and included the same motivational elements as *Life Shift*. The two tracks of intervention included:

1. Brief intervention for low- to at-risk individuals scoring 0 to 15 — The brief intervention focused on the mental and physical driving task, including a) demands on the driving task that are affected by dividing attention between two or more sources of visual information, and b) external (e.g., cell phone use, texting) and internal (e.g., road rage) conditions that affect the driving task. To avoid overlap in content of the *Life Shift* and *Shift Gears* interventions, drunk and drugged driving was not a focus of the control intervention. This interaction between participant and the HE included feedback, problem-solving, exploring the participant’s ambivalence about risky driving and change, and a negotiation/commitment to abstain or reduce risky driving, traffic, or pedestrian behaviors.

2. Referral to treatment for high- to severe-risk individuals scoring 16 to 26+ — In addition to the on-site brief intervention, high- to severe-risk participants received a referral list of free on-line or in-person driving and traffic safety classes.

### Cohort maintenance and follow-up assessment procedures

Pairs of trained bilingual/bicultural MTs, different from the HEs and blind to the participant’s condition, conducted cohort maintenance activities (i.e., phone calls and mailed postcards) and 6-month follow-up assessments. Assessments were typically conducted at a convenient public place selected by the participant, usually at a coffee house. The follow-up visit repeated most of the baseline measures, and a hair sample was collected for those participants reporting abstinence. A $20 incentive at the end of the follow-up visit was provided. Three participants had moved out of the vicinity; therefore, MTs conducted these assessments over the telephone.

### Measures

HEs used standardized instruments, such as the condensed version of the Addiction Severity Index (ASI-Lite), to collect presumed intermediate variables targeted by the intervention; driving and traffic safety attitudes and behaviors; and socio-demographic characteristics during the baseline interview [22]. MTs collected these measures at follow-up. Response cards and machine-scannable surveys were used to facilitate data collection and processing.

### Outcome measures

#### Past 30-day drug abstinence at follow-up

The primary outcome measure was self-reported, past 30-day abstinence measured at 6-month follow-up, a dichotomous variable computed using ASI-Lite. The psychometric properties of the ASI-Lite are similar to those of the longer ASI [30], which is the most widely used assessment tool in the addictions field. The ASI-Lite gathers quantitative information (i.e., number of days in the past 30 days) about the participant’s recent use of several broad types of illicit drugs (heroin, methadone, other opiates/analgesics, barbiturates, other sedatives/hypnotics/tranquilizers, cocaine, amphetamines, cannabis, hallucinogens, and “other drugs”), including alcohol [31,32].

A secondary outcome measure was biologically validated drug use abstinence. Inaccuracies in self-reported drug use post-treatment are likely to be high because of demand characteristics and other factors [33,34]. Therefore, MTs asked for a head hair sample during the follow-up interview to assess past-month use from participants who reported that they had abstained from all illicit drug use in the past 30 days. Samples were collected and hair was segmented to represent the last 30 days of drug use [35]. Hair samples were tested for cocaine, benzoylecgonine, opiates, oxycodone, methamphetamine, amphetamine, and cannabinoids using ELISA (Immunalysis, Pomona, CA) with a detection cut-off of 0.20 nanograms per milligram of hair. Extracts that screened positive were forwarded for confirmational analysis by gas chromatography–mass spectrometry (GC-MS). GC-MS analysis identified and quantified 17 drugs and metabolites: amphetamine, methamphetamine, MDA, MDMA, cocaine, benzoylecgonine, norcocaine, cocaethylene, methadone, codeine, morphine, 6-monoacetylmorphine (heroin metabolite), oxycodone, oxymorphone, hydrocodone, hydromorphone, and meperidine. Instrumentation and methodology were previously published [36]. Cannabinoid results obtained by ELISA were reported qualitatively. The drug analyses conducted, while not comprehensive, tested for the most common drugs of abuse in the County. When hair analysis results were positive for drug use, the individual was considered non-abstinent.

#### Addiction severity index composite scores

Several secondary outcome variables were based on composite scores measured by the ASI-Lite [22]. As mentioned above, the ASI-Lite gathers quantitative information (i.e., number of days in the past 30 days) about the participant’s recent use of several broad types of illicit drugs. In addition, it assesses other areas of the participant’s life commonly affected by substance use: a) medical problems, b) psychiatric problems, and c) alcohol use. Composite scores to measure problem severity were mathematically derived for the four ASI measures by combining responses from each ASI-Lite problem area using recommended scoring procedures [31]. Composite scores range from 0 to 1, with higher scores indicating greater severity of the problem.

#### Health-care utilization

The two hospital sites provided participant-level, health-care utilization data from their cumulative patient databases, which provided additional secondary outcomes. Measures, assessed for the period of 6 months prior and 6 months following the participant’s intervention date, included: a) the number of visits to the ED/trauma unit in the past 6 months, excluding the current visit, b) the number of hospitalizations in the past 6 months, and c) the number of hospital days in the past 6 months. Only one of the two sites was able to provide the last two utilization measures.

#### Driving and traffic safety risk scores

To evaluate the specific effect of the SBIRT *Life Shift* drug intervention in generating positive changes in drug use beyond what attention alone might produce, all participants completed driving and traffic safety attitude and behavior measures at baseline and follow-up. Participants in the driving safety attention-placebo control group were expected to have greater changes in these measures relative to the SBIRT drug use intervention group. An 11-item measure, based partly on the DBQ [27-29], was used to assess changes in driving risks (e.g., texting while driving) as well as general traffic and pedestrian risks (e.g., crossing the street against the light). Participants were asked to indicate how often they performed specific violations/errors based on a 5-point scale ranging from 0 “never” to 5 “nearly all the time”. A mean of the items was computed as an overall score. The internal consistency (Cronbach’s alpha) for the measure was .70.

### Additional variables collected at baseline

Several variables were collected at baseline only to characterize the sample, to ascertain risk levels for intervention intensity, and to serve as covariates in analyses. Sociodemographic variables collected by the HEs at baseline included participants’ gender; age in years; race/ethnicity using the categories Hispanic/Latino, White non-Latino, African-American, and other; and annual household income measured by six categories ranging from less than $9,999 to $50,000 or more. A 4-item self-efficacy scale was used to assess confidence in avoiding drug use in four situations (e.g., when you’re feeling depressed). This brief instrument has shown good validity and reliability with drug users [37]. Items were answered on a Likert scale ranging from 1 to 5, with higher mean scores indicating greater confidence. The internal consistency of the scale was .86. Finally, driving and traffic safety risk scores were used to categorize participants into two groups: low- to at risk, and high- to severe risk.

### Analyses

Chi-square analyses and independent sample t-tests were used to assess the comparability of *Life Shift* and *Shift Gears* participants at baseline, and the degree to which those lost to follow-up were different from those who remained in the study. Abstinence outcomes were analyzed using logistic regression and included site as a covariate. Mixed-model analyses of variance procedures within a general linear model framework were conducted on ASI composite measures and health-care utilization to assess intervention effects. Because ASI composite measures often are not normally distributed, analyses with log-transformed ASI composite scores and utilization data were also conducted. The results did not differ from the non-transformed analyses; consequently, results presented are those of the original analyses. The group-by-time interaction was of particular interest because it is indicative of differential group change. Site was included in all models as a between-subjects factor. Initial tests indicated that follow-up data for the quantitative self-reported outcomes were not missing completely at random. Therefore, two sets of analyses for the self-reported data were conducted: a) complete-case analyses of 292 participants with longitudinal data, and b) intention-to-treat analyses using multiple imputation to deal with loss to follow-up (n = 694) [38]. Imputed outcome values were predicted from baseline drug and alcohol risk levels, gender, age, income, and drug avoidance self-efficacy; imputed values were possible for only 694 participants because six individuals had too many missing data values. Dichotomous abstinence measures at follow-up were analyzed using generalized linear models that controlled for site. Imputation was not possible for categorical outcomes; therefore, analyses of the abstinence outcome are presented for complete cases only. All analyses were conducted using SPSS version 20.

### Sample

#### Participant characteristics

A total of 700 participants were enrolled, with 49.3 percent (n = 346) assigned to the *Life Shift* group, and 50.7 percent (n = 354) assigned to the *Shift Gears* group (see Figure 1). Table 1 presents baseline characteristics of the sample overall and by condition [15]. No significant differences were found between the two groups on any of the baseline characteristics examined. About three-fourths of the participants were men. The average age was in the mid-thirties. The sample was ethnically diverse, with one-third being Latino. Sixty-five percent reported an annual income of less than $10,000 per year. Severity of drug use varied, with about 45 percent at high or severe risk. Ninety-four percent were at relatively low risk for alcohol misuse (i.e., low risk or at risk), not surprisingly, due to the eligibility criteria of greater drug use risk than alcohol use risk. Marijuana was by far the most common drug used (84%). About half of participants were users of marijuana only. A measure of self-efficacy for avoiding drugs was 3.2 on a 5-point scale, ranging from low (1) to high (5) self-efficacy. Approximately 90 percent of participants were in the low- to at-risk level for driving and traffic safety.

**Table 1:** **Characteristics of *Life Shift/Shift Gears *** **participants overall and by condition**

	**Percent or mean (SD)**		
**Characteristic**	**Overall**	***Life Shift***	***Shift Gears***
	**(N = 700)**	**(N = 346)**	**(N = 354)**
Gender (%)			
Male	75.4	76.2	74.6
Female	24.6	23.8	25.4
Age category (%)			
18-20	9.3	10.7	7.9
21-24	13.2	13.6	12.7
25-34	27.1	29.6	24.6
35-44	17.5	16.6	18.4
45-54	22.1	19.5	24.6
55+	10.9	10.1	11.6
Mean age in years	36.9 (13.2)	35.9 (13.3)	37.9 (13.0)
Race/Ethnicity (%)			
Hispanic/Latino	33.1	32.5	33.7
African American	37.0	36.5	37.4
White	24.8	26.0	23.6
Other	5.2	5.0	5.3
Annual income (%)			
Less than $9,999	65.1	64.2	66.1
$10,000 - $14,999	12.9	11.7	13.9
$15,000 - $24,000	8.6	8.1	9.0
$25,000 - $34,999	5.0	6.6	3.5
$35,000 - $49,000	4.0	3.9	4.1
$50,000+	4.4	5.4	3.5
Patient location (%)			
ED	83.5	84.3	82.6
Trauma	16.5	15.7	17.4
Interviewed in Spanish (%)	3.9	4.1	3.7
Drug risk category (%)^a^			
At risk	55.1	55.0	55.2
High risk	41.1	40.6	41.7
Severe risk	3.8	4.4	3.2
Alcohol use risk category (%)^b^			
Low risk	70.3	73.7	67.0
At risk	24.0	21.6	26.5
High risk	4.3	3.3	5.4
Severe risk	1.3	1.5	1.2
Type of drug used (%)^c^			
Marijuana	84.4	87.5	81.4
Amphetamines	19.3	17.4	21.2
Cocaine	8.8	7.8	9.7
Heroin	7.8	6.1	9.6
Other opiates	7.4	7.6	7.3
User of Marijuana only (%)	49.6	51.6	47.7
Use of more than 1 substance incl. alcohol (%)	49.9	47.9	51.9
Mean drug avoidance self-efficacy score	3.2 (1.19)	3.2 (1.2)	3.3 (1.2)
Driving and traffic safety risk category (%)			
Low- and at risk	91.3	93.4	89.2
High- and severe risk	8.7	6.7	10.6

^a^Based on the Drug Abuse Screening Test — DAST-10 [19,20]. Risk categories included at risk (score of 1–2), high risk (score of 3–8), and severe risk (score of 9–10).

^b^Based on the Alcohol Use Disorders Identification Test — AUDIT [18]. Risk categories included no/low risk (score of 0–7), at risk (score of 8–15), high risk (score of 16–19), and severe risk (score of 20–40).

^c^Multiple drug use was possible. Therefore, the total percent exceeds 100%.

#### Loss to follow-up

The overall follow-up rate was 42 percent (292 of the 700 were followed successfully). Figure 1 presents the reasons for loss to follow-up by condition. Analyses indicated that dropout status was not related to condition, site, gender, race/ethnicity, income, driving/traffic risk scores, baseline alcohol use risk level, or the baseline medical, psychiatric, or alcohol use composite scores. However, younger people, those with more severe baseline drug use, and those with lower baseline drug use avoidance self-efficacy were more likely to be lost to follow-up.

## Results

### Participation in hair sampling at follow-up

Thirty-one participants at follow-up reported being abstinent from all drugs during the past 30 days. MTs collected hair samples for verification from 14 of the 31 participants (8 *Life Shift* and 6 *Shift Gears* participants). Of the 14 that provided hair samples, 10 were found to be abstinent and four were non-abstinent. Hair samples were not collected for the remaining 17 participants for the following reasons: a) nine declined to provide a hair sample at the time of follow-up, b) three were not available because they had moved out of the state and conducted the interview with the MT by telephone, c) three had insufficient body hair, and d) two failed to have hair samples taken for unknown reasons. Missing collection of hair among those reporting abstinence did not differ by condition. Conservatively, all 17 were assumed to be non-abstinent in the analysis of past 30-day abstinence.

### Effects of intervention

As shown in Table 2, self-reported past 30-day abstinence from all drug use assessed during the follow-up visit was 12.5 percent for *Life Shift* and 12 percent for *Shift Gears*, a non-significant difference (p = .888). When results of hair analyses were applied, abstinence rates were 7 percent for *Life Shift* and 2 percent for *Shift Gears;* the difference between groups was not significant (p = .074).

**Table 2:** **Outcomes of *Life Shift/Shift Gears *** **using complete cases (n=292) and imputed cases (n = 694)**

**Means (SE) or percent**					
**Outcome**	***Life Shift *** **intervention**		***Shift Gears *** **control**		**p value for group ×; time interaction or Wald χ**^**2**^
	**Baseline**	**Follow-up**	**Baseline**	**Follow-up**	
Past 30 day drug abstinence at follow-up (%)^b^					
Self-reported		12.5 (3.2)		12.0 (3.1)	.888
Biologically validated^c^		7.0 (2.5)		2.0 (1.2)	.074
Self-reported drug use – ASI Composite Score (0-1)					
Complete cases	.059 (.008)	.068 (.010)	.055 (.007)	.095 (.010)	.035^a^
Imputed	.070 (.005)	.075 (.006)	.068 (.006)	.085 (.007)	.124^a^
Medical problems – ASI Composite Score (0-1)					
Complete cases	.639 (.021)	.176 (.038)	.696 (.020)	.280 (.036)	.404^a^
Imputed	.65 (.013)	.219 (.023)	.669 (.014)	.248 (.024)	.627^a^
Psychiatric problems – ASI Composite Score (0-1)					
Complete cases	.287 (.031)	.250 (.029)	.292 (0.27)	.243 (.025)	.734^a^
Imputed	.264 (.017)	.239 (.016)	.272 (.017)	.228 (.017)	.404^a^
Alcohol use – ASI Composite Score (0-1)					
Complete cases	.127 (.018)	.124 (.016)	.106 (.017)	.106 (.015)	.888
Imputed	.126 (.011)	.115 (.009)	.126 (.012)	.112 (.010)	.808
Past 6 mo. health care utilization					
No. of ED visits^d^	.528 (.106)	.828 (.129)	.549 (.111)	.806 (.135)	.826^a^
No. of hospitaliz.^e^	.196 (.094)	.261 (.111)	.146 (.099)	.317 (.117)	.479
No. of days hosp.^e^	1.26 (.607)	1.60 (643)	1.43 (.643)	2.56 (.681)	.335^a^
Driving and traffic risk scores					
Complete cases	.975 (.068)	.831 (.070)	.840 (.064)	.907 (.066)	.057
Imputed	1.00 (.045)	.935 (.042)	.837 (.048)	.897 (.045)	.165

^a^Significant time main effect, p < .05.

^b^Imputation not available for dichotomous outcomes, therefore, analyses based on 292 complete cases.

^c^Those followed and reporting 0 drug use but who did not provide a hair sample for any reason; assumed to be nonabstinent.

^d^Data available for 602 patients.

^e^Site 2 only; data available for 97 patients.

Table 2 presents mean ASI drug use composite scores by condition and results of statistical tests of changes. Of interest are the relatively low baseline drug use composite scores in both groups (approximately .06 on a 0 to 1 scale). Among complete cases, the *Life Shift* intervention group showed relatively small increases in ASI composite scores for self-reported use of any drugs compared to the *Shift Gears* attention-placebo control group. The differential change resulted in a significant group-by-time interaction (p = .035) in favor of *Life Shift* intervention effectiveness, in addition to a time main effect. An additional analysis indicated that the differential group change in drug use scores did not differ for marijuana-only users versus users of other drugs (data not shown). When imputed data were analyzed, however, the interaction was no longer statistically significant.

Psychiatric problems, and particularly medical problems, declined over time in *both* groups at about the same rate, resulting in a statistically significant time main effect that was found with complete cases and imputed data. Alcohol use showed no time main effect or interaction. Considering health-care utilization outcomes, there were no group-by-time interactions; however, both groups increased over time (time main effect) in the number of ED visits and the number of hospital days. Number of hospitalizations also increased in both groups, but did not approach statistical significance.

Results of driving and traffic risk scores showed a marginally significant interaction effect (p = .057), with the *Life Shift* intervention participants showing greater improvement than *Shift Gears* participants. This finding was unexpected insofar as those in the *Shift Gears* condition received the intervention in reducing driving and traffic risks, whereas the *Life Shift* group did not receive that intervention.

## Discussion and conclusions

This study found no support for the effectiveness of the SBIRT approach for illicit drug use. The primary outcome variable, past 30-day drug abstinence, was not significant. Analyses of ASI drug use composite scores using imputed data were also not significant.

Comparing our results to those of others is difficult given the lack of comparable study designs, differences in the types of drug users targeted, and other important methodological differences. Bernstein and colleagues’ randomized trial of brief motivational intervention in clinics for opioid and cocaine users is the most similar to the present study in terms of design [13]. Those authors reported a 4.6 percentage-point difference in biologically validated past 30-day abstinence rates between intervention and control groups at 6-month follow-up [13], similar to the 5 percentage-point difference in abstinence rates reported here. They also reported beneficial effects of the brief intervention on ASI drug and medical composite scores. Their results are in stark contrast to ours, insofar as we did not see reductions in ASI drug scores in the SBIRT intervention group. Differences in enrollment criteria, the racial/ethnic composition of participants, the content/intensity of what the control group received, and the type of drug users enrolled make formal comparison between the two study results difficult. It is also noteworthy that the Bernstein study enrollees had much higher ASI drug use scores at baseline (.25 versus .06), and lower ASI medical scores (.56 versus .67) than did our participants. Perhaps the benefits of the SBIRT approach are more greatly realized among those at higher addiction levels.

Our study sample differed from those reported elsewhere in terms of their ASI scores. For example, the drug use composite score in our sample of .056 is lower than the score of .09 reported for a nationally representative sample of those in outpatient treatment programs [32], and far lower than the score of .25 reported for opioid/cocaine users [13]. These differences are not surprising given that the present study sample was not in treatment; so one would expect them to have lower composite scores. The current study had a large proportion of marijuana-only users; to the degree that they were less likely to perceive their drug use as a problem due to changing societal norms, their drug use composite scores would be relatively low. Psychiatric problem scores in the current sample, however, were almost twice as high as those among patients undergoing outpatient drug treatment [32], and medical problem scores were 24 percent higher than among opiate and cocaine users [13]. These differences underscore the heterogeneity of drug users in terms of their co-morbid mental health and medical status, and underscore the importance of addressing mental health and/or medical needs within a population needing substance use treatment.

There was no evidence that the SBIRT drug intervention had an effect on medical/ psychiatric problems, alcohol use, or health-care utilization. Although results from other studies are mixed, our findings are in line with a meta-analysis that found no statistically significant effect of SBIRT interventions on health-care utilization [39]. Furthermore, the U.S. Preventive Services Task Force [40] reported that evidence is insufficient to demonstrate that psychosocial intervention reliably improves non-drug use outcomes for largely asymptomatic patients whose illicit drug use is detected through screening. Time main effects for several of these outcomes were observed in the present study, indicating similar changes in both intervention and control groups. For example, there were decreases in self-reported medical and psychiatric problems often associated with drug use, and increases in health-care utilization for both groups. Although this pattern might seem paradoxical, differences in the measures may partly explain the finding. ASI medical and psychiatric problem scores targeted problems in the past 30 days, whereas health-care utilization measures covered a longer period of time (past 6 months). Furthermore, participants’ medical and psychiatric problems at baseline were likely relatively high, insofar as they were currently patients in an ED visiting for a medical problem. At follow-up, however, there were no patients awaiting care. Researchers suggest that patients may no longer have been troubled by their previous medical problems, and therefore, their problem scores were lower.

Despite cohort maintenance activities, a high dropout rate was a limitation of the study. In the present study, populations more likely to be lost to follow-up included younger people, individuals with more severe baseline drug use, and those with lower baseline drug use avoidance self-efficacy. High dropout is a reported problem in many drug use studies, although other studies similar to the present one have achieved high follow-up rates [11,13]. Low participation in hair collection for confirming drug abstinence was also a limitation. Another possible limitation is related to using hair samples to confirm self-reported drug use at follow-up, but not at baseline. Participants were expected to have accurate disclosure at admission into the study [16,41], although others have reported that individuals may report drug use at baseline that is not confirmed [13]. In addition, because the HE delivered the type and level of intervention, he/she was aware of the participant’s assigned condition, so some bias may have been introduced. However, measurement staff members were blind to the participants’ conditions. Finally, a small sample size (n = 97) for some analyses was also a problem.

The methodology of the study has many strengths, including its minimal exclusion criteria. The decision to include participants who were using an assortment of substances and multiple substances was made for both scientific and clinical reasons. Recruitment of varied and polydrug users in research trials has been advocated by experts in the field of substance use disorder research as a “real world” test of an approach, and as a means to bridge the gap between research and practice [42]. The use of an attention-placebo control group is also a strength, as it allowed us to test the rival hypothesis that improvement in drug use occurred because of the participant’s expectations or the attention received, rather than from the SBIRT itself [43]. Unexpectedly, the SBIRT group tended to improve more than the placebo group on the measure related to driving and traffic safety. The SBIRT drug use intervention may have been more salient or interesting than the attention-placebo control intervention, bringing about changes in a variety of health and safety areas among those participants who received it. Attempted biochemical validation of reports of abstinence, a multiethnic sample, and rigorous modeling of missing data are additional strengths.

The null results of the present study are disappointing, yet it is premature to conclude that SBIRT cannot work for drug use. Alternative explanations, such as those related to intervention implementation and measurement may have obfuscated SBIRT’s effects. Future studies are needed to rule out alternative explanations and add to the knowledge about SBIRT effectiveness for drug use.

## Competing interests

The authors declare that they have no competing interests.

## Authors’ contributions

SW and JC contributed to the conceptualization of the manuscript. SW, KE, and CM participated in data analyses. KE, MH, AS, CBS, EC, TC, and MS participated in the implementation and intervention aspects of the study. JG conducted the hair analyses. All authors read and approved the final manuscript.

## References

[B1] San Diego Association of Governments (SANDAG) Reports reveal trends in drug abuse & gang involvementhttp://www.sandag.org/index.asp?newsid=730&fuseaction=news.detail

[B2] County of San Diego, Health and Human Services Administration, Behavioral Health Facts and statistics about drug and alcohol abusehttp://sandiego.networkofcare.org/mh/library/article.aspx?id=393

[B3] Cherpitel CJ Ye Y Alcohol-attributable fraction for injury in the U.S. general population: data from the 2005 National alcohol survey J Stud Alcohol Drugs 2005 9 535 538 10.15288/jsad.2008.69.535 18612569

[B4] Substance Abuse and Mental Health Services Administration (SAMHSA)/Center for Substance Abuse Treatment (CSAT) A guide to substance abuse services for primary care clinicians Treatment Improvement Protocol (TIP) Series 24 1997 Rockville: U.S: Department of Health and Human Services, Substance Abuse and Mental Health Services Administration DHHS Publication No. (SMA) 97–3139 22514830

[B5] Babor TF McRee BG Kassebaum PA Grimaldi PL Ahme K Bray J Screening, brief intervention, and referral to treatment (SBIRT): toward a public health approach to the management of substance abuse Subst Abus 2007 9 3 7 30 10.1300/J465v28n03_03 18077300

[B6] Longabaugh R Woolard RF Nirenberg TD Minugh AP Becker B Clifford PR Carty K Licsw Sparadeo F Gogineni A Evaluating the effects of a brief motivational intervention for injured drinkers in the emergency department J Stud Alcohol 2001 9 806 816 11838918 10.15288/jsa.2001.62.806

[B7] Wilk AI Jensen NM Havighurst TC Meta-analysis of randomized control trials addressing brief interventions in heavy alcohol drinkers J Gen Intern Med 1997 9 5 274 283 10.1007/s11606-006-5063-z 9159696 PMC1497107

[B8] D’Onofrio G Degutis LC Preventive care in the emergency department: screening and brief intervention for alcohol problems in the emergency department: a systematic review Acad Emerg Med 2002 9 627 638 10.1111/j.1553-2712.2002.tb02304.x 12045080

[B9] Saitz R Screening and brief intervention enter their 5th decade Subst Abus 2007 9 3 3 6 10.1300/J465v28n03_02 18077299

[B10] Saitz R Alford DP Bernstein J Cheng DM Samet J Palfai T Screening and brief intervention for unhealthy drug use in primary care settings: randomized clinical trials are needed J Addict Med 2010 9 3 123 130 10.1097/ADM.0b013e3181db6b67 20936079 PMC2950314

[B11] Humeniuk R Ali R Babor T Souze-Formigoni ML de Lacerda RB Ling W McRee B Newcombe D Pal H Poznyak V Simon S Vendetti J A randomized controlled trial of a brief intervention for illicit drugs linked to the alcohol, smoking and substance involvement screening test (ASSIST) in clients recruited from primary health-care settings in four countries Addiction 2012 9 957 966 10.1111/j.1360-0443.2011.03740.x 22126102

[B12] Madras BK Compton WM Avula D Stegbauer T Stein JB Clark HW Screening, brief interventions, referral to treatment (SBIRT) for illicit drug and alcohol use at multiple healthcare sites: comparison at intake and 6 months later Drug Alcohol Depend 2009 9 280 295 10.1016/j.drugalcdep.2008.08.003 18929451 PMC2760304

[B13] Bernstein J Bernstein E Tassiopoulos K Heeren T Levenson S Hingson R Brief motivational intervention at a clinic visit reduces cocaine and heroin use Drug Alcohol Depend 2005 9 49 59 10.1016/j.drugalcdep.2004.07.006 15607841

[B14] Bernstein E Bernstein JA Stein JB Saitz R SBIRT in emergency care settings: are we ready to take it to scale? Acad Emerg Med 2009 9 1072 1077 10.1111/j.1553-2712.2009.00549.x 20053225

[B15] Eisenberg K Woodruff SI Randomized controlled trial to evaluate screening and brief intervention for drug-using multiethnic emergency and trauma department patients Addict Sci Clin Pract 2013 9 8 doi:10.1186/1940-0640-8-8 10.1186/1940-0640-8-8 23566363 PMC3642029

[B16] Magura S Validating self-reports of illegal drug use to evaluate national drug control policy: a reanalysis and critique Eval Program Plann 2010 9 234 237 10.1016/j.evalprogplan.2009.08.004 19765827

[B17] Nutt DJ King LA Phillips LD Independent Scientific Committee on Drugs: drug harms in the UK: a multicriteria decision analysis Lancet 2010 9 1558 1565 10.1016/S0140-6736(10)61462-6 21036393

[B18] Babor TF Higgins-Biddle JC Saunders JB Monteiro MG AUDIT – The Alcohol Use Disorders Identification Test: Guidelines for Use in Primary Care 2nd Edition 2001 Geneva, Switzerland: World Health Organization, Department of Mental Health and Substance Dependence

[B19] Yudko E Lozhkina O Fouts A A comprehensive review of the psychometric properties of the drug abuse screening test J Subst Abuse Treat 2007 9 189 198 10.1016/j.jsat.2006.08.002 17306727

[B20] Skinner HA The drug abuse screening test Addict Behav 1982 9 4 363 371 10.1016/0306-4603(82)90005-3 7183189

[B21] Moher D Schulz KF Altman DG CONSORT group: the CONSORT statement: revised recommendations for improving the quality of reports of parallel-group randomized trials BMC Med Res Methodol 2001 9 2 10.1186/1471-2288-1-2 11336663 PMC32201

[B22] McLellan AT Luborsky L Woody GE O’Brien CP An improved diagnostic evaluation instrument for substance abuse patients: the addiction severity index J Nerv Ment Dis 1980 9 1 26 33 10.1097/00005053-198001000-00006 7351540

[B23] Murphy SA Collins LM Rush AJ Editorial: customizing treatment to the patient: adaptive treatment strategies Drug Alcohol Depend 2007 9 2 S1 S3 17350181 10.1016/j.drugalcdep.2007.02.001 PMC1924645

[B24] Prochaska JO DiClemente CC Norcross JC In search of how people change: applications to addictive behaviors Am Psychol 1992 9 9 1102 1114 1329589 10.1037//0003-066x.47.9.1102

[B25] Miller WR Motivational interviewing with problem drinkers Behav Psychother 1983 9 147 172 doi: http://dx.doi.org/10.1017/S0141347300006583 10.1017/S0141347300006583

[B26] Miller WR Rollnick S Motivational interviewing: preparing people for change (2nd ed) 2002 New York: Guilford Press

[B27] Lawton R Parker D Manstead ASR Stradling SG The role of affect in predicting social behaviors: the case of road traffic violations J Appl Soc Psychol 1997 9 14 1258 1276 doi: 10.1111/j.1559-1816.1997.tb01805.x 10.1111/j.1559-1816.1997.tb01805.x

[B28] Parker D Reason JT Manstead ASR Stradling SG Driving errors, driving violations and accident involvement Ergonomics 1995 9 5 1036 1048 doi: 10.1080/00140139508925170 10.1080/00140139508925170 29105607

[B29] Reason JT Manstead ASR Stradling SG Baxter JS Campbell K Errors and violations on the road: a real distinction? Ergonomics 1990 9 1315 1332 doi: 10.1080/00140139008925335 10.1080/00140139008925335 20073122

[B30] Cacciola JS Alterman AI McLellan AT Lin YT Lynch KG Initial evidence for the reliability and validity of a “lite” version of the addiction severity index Drug Alcohol Depend 2007 9 297 302 17045423 10.1016/j.drugalcdep.2006.09.002

[B31] McGahan PL Griffith JA Parente R McLellan AT: Composite scores for the addiction severity index 1986 Philadelphia, PA: Department of Veterans Affairs Medical Center Retrieved from http://www.tresearch.org/wp-content/uploads/2012/09/

[B32] McLellan AT Cacciola JC Alterman AI Rikoon SH Carise C The addiction severity index at 25: origins, contributions and transitions Am J Addict 2006 9 2 113 124 10.1080/10550490500528316 16595348

[B33] Victorin K Haag-Gronlund M Skerfving S Methods for health risk assessment of chemicals: are they relevant for alcohol? Alcohol Clin Exp Res 1998 9 7 270S 276S 10.1111/j.1530-0277.1998.tb03923.x 9799949

[B34] Wish ED Hoffman JA Nemes S Harrison , Hughes A The validity of self-reports of drug use at treatment admission and at follow-up: comparisons with urinalysis and hair assays Validity of Self-Reported Drug Use: Improving the Accuracy of Survey Estimates, Research Monograph 167L 1997 Rockville: National Institute on Drug Abuse 200 226 NIH Publication No. 97–4147 9243563

[B35] Pragst F Balikova MA State of the art in hair analysis for detection of drug and alcohol abuse Clin Chim Acta 2006 9 17 49 10.1016/j.cca.2006.02.019 16624267

[B36] Aleksa K Walasek P Fulga N Kapur B Gareri J Koren G Simultaneous detection of seventeen drugs of abuse and metabolites in hair using solid phase micro extraction (SPME) with GC/MS Forensic Sci Int 2012 9 1–3 31 36 22047752 10.1016/j.forsciint.2011.10.002

[B37] Coon GM Pena D Illich PA Self-efficacy and substance abuse: assessment using a brief phone interview J Subst Abuse Treat 1998 9 5 385 391 10.1016/S0740-5472(97)00285-7 9750996

[B38] Rubin RB Multiple Imputation for Nonresponse in Surveys 1987 New York: John Wiley and Sons, Inc.

[B39] Bray JW Cowell AJ Hinde JM A systematic review and meta-analysis of health care utilization outcomes in alcohol screening and brief intervention trials Med Care 2011 9 3 287 294 doi: 10.1097/MLR.0b013e318203624f 10.1097/MLR.0b013e318203624f 21263359 PMC4691530

[B40] Polen MR Whitlock EP Wisdom JP Nygren P Bougatsos C Screening in primary care settings for illicit drug use: staged systematic review for the United States preventive services task force. Evidence Synthesis No. 58, Part 1 2008 Agency for Healthcare Research and Quality: Rockville 20722153

[B41] Hinden R McCusker J Vickers-Lahti M Bigelow C Garfield F Lewis B Radioimmunoassay of hair for determination of cocaine, heroin, and marijuana exposure: comparison with self-report Int J Addict 1994 9 6 771 789 8034385 10.3109/10826089409047909

[B42] Rounsaville BJ Petry NM Carroll KM Single versus multiple drug focus in substance abuse clinical trials research Drug Alcohol Depend 2003 9 2 117 125 10.1016/S0376-8716(03)00033-4 12732403 PMC3662471

[B43] Bootzin RR White L, Tursky B, Schwartz GE The role of expectancy in behavior change Placebo: Theory, Research and Mechanisms 1985 New York: Guilford Press 196 210

